# Secretome Analysis Performed During *in vitro* Cardiac Differentiation: Discovering the Cardiac Microenvironment

**DOI:** 10.3389/fcell.2020.00049

**Published:** 2020-02-07

**Authors:** Anny Waloski Robert, Isabela Tiemy Pereira, Bruno Dallagiovanna, Marco Augusto Stimamiglio

**Affiliations:** Laboratório de Biologia Básica de Células-Tronco, Instituto Carlos Chagas – Fiocruz-Paraná, Curitiba, Brazil

**Keywords:** secretome, extracellular matrix, cardiac differentiation, embryonic stem cells, conditioned medium

## Abstract

Human pluripotent stem cells are an important tool for the study of developmental processes, such as cardiomyogenic differentiation. Despite the advances made in this field, the molecular and cellular signals involved in the commitment of embryonic stem cells to the cardiac phenotype are still under investigation. Therefore, this study focuses on identifying the extracellular signals involved in *in vitro* cardiac differentiation of human embryonic stem cells. Using a three-dimensional cardiomyogenic differentiation protocol, the conditioned medium and the extracellular matrix (ECM) of embryoid body cultures were collected and characterized at four specific time points. Mass spectrometry (MS) and antibody array analysis of the secretome identified a number of secreted proteins related to signaling pathways, such as Wnt and TGFβ, as well as many ECM proteins. When comparing the proteins identified at selected time points, our data pointed out protein interactions and biological process related to cardiac differentiation. Interestingly, the great changes in secretome profile occurred during the cardiac progenitor specification. The secretome results were also compared with our previous RNAseq data, indicating that the secreted proteins undergo some level of gene regulation. During cardiac commitment it was observed an increase in complexity of the ECM, and some proteins as IGFBP7, FN1, HSPG2, as well as other members of the basal lamina could be highlighted. Thus, these findings contribute valuable information about essential microenvironmental signals working on cardiomyogenic differentiation that may be used in future strategies for cardiac differentiation, cardiomyocyte maturation, and in advances for future acellular therapies.

## Introduction

*In vitro* cardiomyogenic differentiation of human embryonic (hESC) or induced pluripotent stem cells (hiPSCs) is an alternative to modeling two basic research studies: (a) enables the study of cardiomyogenesis process, cardiac disease modeling, and development of cardiotoxicity tests, and (b) can be used in cell therapy protocols, thereby generating specific cell populations, e.g., cardiomyocytes for tissue replacement (Murry and Keller, 2008; Van Vliet et al., 2012; Musunuru et al., 2018). The commitment to the cardiac lineage during embryonic development includes several steps that are regulated by a network of transcription factors and signaling pathways that control the specification and maturation of cardiac cells (Olson, 2006; Van Vliet et al., 2012). Based on these observations, the literature presents numerous protocols for *in vitro* differentiation through the modulation of well-known signaling pathways (Elliott et al., 2011; Kattman et al., 2011; Lian et al., 2013). Despite these findings, the complete comprehension of the molecular basis of cardiomyogenic differentiation is still not well understood.

To characterize the genes, signaling pathways and regulatory networks involved in this cell differentiation process, large-scale studies have been performed. Genome-wide expression profiling using microarray or RNA sequencing was performed at different stages of *in vitro* cardiac differentiation (Xu et al., 2009; Hartogh et al., 2016; Liu et al., 2017; Friedman et al., 2018; Fu et al., 2018), and more recently, the importance of posttranscriptional gene regulation was also highlighted (Garate et al., 2018; Pereira et al., 2019). Global proteome analysis of cardiomyocyte differentiation has been reported with different methodologies, but they described whole cell or membrane proteomes (Van Hoof et al., 2010; Poon et al., 2015; Hofsteen et al., 2016; Konze et al., 2017a, b) with little emphasis on the secreted proteins, such as those of the extracellular matrix (ECM). Thus, despite the importance of extracellular signaling in cellular behavior and differentiation, few studies have investigated the secreted factors released during cardiac differentiation.

The set of proteins secreted by cells, including soluble factors, ECM, and proteins present in microvesicles/exosomes, comprise the secretome. In 2010, Stastna et al. (2010) compared the secretome of rat cardiac stem cells with cardiomyocytes from neonatal mice and identified 53 proteins described as membrane or secreted proteins. Then, the characterization of conditioned medium (CM) of mouse ESC at two time points of spontaneous cardiac differentiation identified approximately 150 proteins (Farina et al., 2011). Considering specifically the secretome of cardiac progenitors, either from mouse or human, various cytokines, growth factors, and other proteins were described in the CM and microvesicles of these cells (Zhang et al., 2015; Park et al., 2016; Nie et al., 2018; Samal et al., 2019). As an example, Sharma et al. (2017) isolated human cardiac progenitor cells (CPCs) from newborns and adults and compared the composition and functionality of their respective CMs. This proteomic analysis identified several proteins (between 500 and 800) that revealed differences in the composition and the effect of the secretome obtained from cells at different ages, indicating that during development, the cells change their secretion profile (Sharma et al., 2017).

In fact, the cardiogenic niche is highly dynamic, presenting different functionalities and characteristics according to the stage of heart development and its physiological state (Lockhart et al., 2011). Therefore, investigating differences in the secretome during development could be a means of obtaining useful information, not only to improve the knowledge about cardiomyogenesis but also to identify key factors with potential clinical use. Recently, Wolling et al. (2018) characterized the CM of hESCs and hiPSCs during cardiac differentiation using a monolayer differentiation protocol. The in-depth proteomic analysis revealed that more than 1800 proteins were regulated among the 7 analyzed time points, and among them, 431 were found to be secreted proteins (Wolling et al., 2018). Despite these advances, 3D modeling studies have highlighted the importance of holding on the contact and communication between cells, which clearly modulates microenvironmental signaling (Shamir and Ewald, 2014). Hence, studies comparing distinct cell differentiation protocols and using 2D and 3D cell culture approaches are desirable to better comprehend cell signaling during differentiation.

In addition to the soluble factors secreted by cells in the tissue niche, the ECM functions as cell scaffolding, influences cell behavior and fate (Rozario and DeSimone, 2010) and plays an important role in cell signaling. Although the characterization of the CM allows the identification of some ECM proteins, tissue decellularization methodologies were developed for a better characterization of ECM molecules from various types of tissues and organs, including the heart (Ott et al., 2008; Lu et al., 2013). Nevertheless, the constitution of the ECM during different phases of *in vitro* cardiac differentiation has not yet been elucidated. We considered that knowledge about the cardiac microenvironment will not only generate a better understanding of the cell differentiation process but also help to create new strategies to improve the efficiency of the process and to optimize *in vitro* cardiomyocyte maturation. Additionally, soluble factors or ECM proteins may be used in the future as a cell-free therapy to treat cardiovascular diseases.

Thus, the aim of this work was to focus on the extracellular signals secreted by hESCs during the process of cardiac differentiation. To this end, we isolated and characterized both soluble factors from the culture (CM) and the ECM during the *in vitro* cardiomyogenic differentiation of hESCs. We also analyzed our previous data on polysome mRNA profiling (Pereira et al., 2018) to identify extracellular signals upregulated during cardiomyogenesis and compared them with our proteomic results.

## Materials and Methods

### Cell Culture and Cardiomyogenic Differentiation

The hESC lineage used in this work was the HES3 NKX2-5^eGFP/w^ (Elliott et al., 2011), which was generously provided by Monash University (Victoria, Australia). hESCs were cultured on a feeder layer of irradiated mouse embryonic fibroblasts in Dulbecco’s modified Eagle’s medium (DMEM)/F12 supplemented with 20% KnockOut^TM^ serum replacement (KSR, Gibco^TM^), 100 U/ml penicillin, 100 μg/ml streptomycin, 2 mM L-glutamine, 1% non-essential amino acid, 0.1 mM β-mercaptoethanol, and 10 ng/ml human basic fibroblast growth factor (βFGF) (Sigma). The medium was changed daily until cultures reached 80–90% confluence; at this point, cells were dissociated using 0.05% trypsin/EDTA.

**FIGURE 1:**
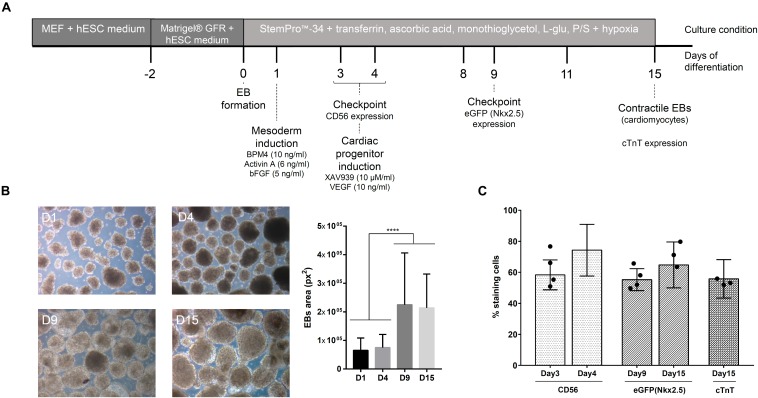
Cardiomyogenic differentiation of hESCs. **(A)** Timeline of the cardiac differentiation protocol. **(B)** Representative images of embryoid bodies (EBs) during differentiation and the measurement of their size (manual measurement at ImageJ software) (*n* = 10 images). ****p-value < 0.0001; statistical analysis was performed using one-way ANOVA and Tukey’s posttest. **(C)** Percentage (mean ± SD) of cells positive for different markers during cardiac differentiation (CD56 – mesoderm; eGFP/Nkx2.5 – cardiac progenitor; cTnT – cardiomyocytes) (*n* = 6); the black dots represent the percentage of respective markers in cell population selected for experiments used for MS analysis.

The cardiomyogenic differentiation protocol used was first described by Kattman et al. (2011) and previously standardized in our group (Pereira et al., 2018). The protocol steps are represented in Figure 1A. During the differentiation protocol, the basal medium used was composed of StemPro^TM^-34, supplemented with transferrin (150 μg/ml), ascorbic acid (50 μg/ml), monothioglycerol (0.45 mM), L-glutamine (2 mM), and penicillin (100 U/ml)/streptomycin (100 μg/ml). Briefly, after embryoid body (EB) formation (day 1, D1), the EBs were induced to mesodermal commitment by stimulation with BMP4 (10 ng/ml), Activin A (6 ng/ml), and βFGF (5 ng/ml) in culture medium. On day 4 (D4), the cells were immunophenotyped to CD56, a mesodermal marker (Evseenko et al., 2010), to verify the expression of this glycoprotein in the cell population. Next, EBs were induced to cardiac progenitor differentiation in the presence of XAV939 (10 μM/ml) and VEGF (10 ng/ml) in medium followed by confirmation of the cell phenotype on day 9 (D9) through eGFP/Nkx2.5 expression. From day 8 (D8) onward, the EBs were maintained in medium supplemented with VEGF (10 ng/ml), and at day 15 (D15), the EBs were dissociated and immunophenotyped to check the expression of cardiac troponin T (cTnT), a marker for cardiomyocytes. The percentage of cells cTnT^+^ in the culture was related to the efficiency of the cardiac differentiation protocol.

### Preparation of Conditioned Medium and ECM for Mass Spectrometry

Human embryonic stem cells were submitted to cardiomyogenic differentiation as described above, and the CM from cultures were collected on days 1 (CMd1), 4 (CMd4), 8 (CMd8), and 15 (CMd15) of the protocol (coincident with culture medium exchange days). After collection, the CMs were centrifuged at 1,620 × *g* for 5 min and then at 4,000 × *g* for 20 min at 8°C to remove cell debris and major apoptotic bodies. As a control, the basal medium (StemPro^TM^-34 without any growth factor) was used (named as non-CM – nCM). Protein quantification was determined by a Qubit^®^ Protein Assay kit (Molecular Probes) following the manufacturer’s instructions. Samples were stored at −80°C until use.

For each time point of the differentiation protocol, CM from three independent assays was collected. Approximately 70 μg of protein from samples was mixed with SDS sample buffer (160 mM Tris–HCl pH 6.8, 4% SDS, 10% β-mercaptoethanol, 24% glycerol, and 0.02% bromophenol blue), and the CM samples were resolved in 10% SDS-PAGE. Initial SDS-PAGE and mass spectrometry (MS) analysis revealed that the bands between 50 and 75 kDa were predominantly composed of albumin and serotransferrin (Supplementary Figure 1A). Then, to decrease the presence of these contaminant proteins, in all the SDS-PAGE samples, the region between 50 and 75 kDa was excised and excluded before MS.

To obtain the ECM secreted by cells during cardiomyogenic differentiation, EBs were decellularized as previously described (Goh et al., 2013; Sart et al., 2014). Briefly, EBs at days 1 (ECMd1), 4 (ECMd4), 9 (ECMd9), and 15 (ECMd15) were collected and submitted to treatment with 1% Triton X-100 for 30 min under stirring followed by centrifugation at 18,000 × *g* for 2 min. Then, samples were treated with DNAse I (1 mg/ml) for 30 min at room temperature, centrifuged, and rinsed twice with PBS. Two experimental replicates from each time point of the protocol were obtained. Decellularization was confirmed by DNA extraction with the QIAamp DNA mini kit (Qiagen) following the manufacturer’s instructions. Decellularized EBs were solubilized in 50 μl of SDS sample buffer, resolved in 10% SDS-PAGE, and analyzed by MS.

### LC–MS/MS Analysis and Protein Identification

The SDS-PAGE lanes were excised and sliced for in-gel tryptic digestion of the proteins. After trypsinization, the obtained peptides were extracted from the gel matrix, concentrated in a vacuum centrifuge, and desalted with homemade C18 spin columns. Five micrograms of proteins were analyzed in duplicate (for CMs and nCM samples) or in triplicate (for ECM samples) by LC–MS/MS in a Thermo Scientific Easy-nLC 1000 system coupled to an LTQ Orbitrap XL ETD (MS facility RPT02H/Carlos Chagas Institute – Fiocruz Parana). Peptides were separated on a 30-cm-long (75 μm inner diameter) fused silica in-house packed with reversed-phase ReproSil-Pur C18-AQ 1.9 μm resin (Dr. Maisch GmbH, Ammerbuch-Entringen, Germany) using a 120 min gradient (from 5 to 40% MeCN in 0.1% formic acid, 5% DMSO) at a flow rate of 250 nl/min. Survey full-scan MS spectra (at 300–2,000 *m/z* range) were acquired in an Orbitrap analyzer with a resolution of 60,000 at *m/z* 400. The 10 most intense ions were sequentially isolated and fragmented in the linear ion trap using collision-induced dissociation. The “lock mass” option was enabled in all full scans to improve the mass accuracy of precursor ions (Olsen et al., 2005).

Raw files were uploaded into the MaxQuant platform (versions 1.5.8.3 and 1.6.1.0) (Cox and Mann, 2008) using the algorithm Andromeda (Cox et al., 2011) for searching proteins against a human protein sequence database (UniProt, *Homo sapiens*, 70.939 and 71.599 entries, downloaded in May 2017 and May 2018, respectively). Search parameters specified an MS tolerance of 4.5 ppm, an MS/MS tolerance of 0.5 Da, and full trypsin specificity, enabling up to two missed cleavages. Cysteine carbamidomethylation was set as a fixed modification and oxidation of methionines and N-terminal acetylation of protein as variable modifications. For validation of the identifications, a minimum of seven amino acids for peptide length and two peptides per protein was required. In addition, a false discovery rate (FDR) of 1% was applied at both the peptide and protein levels. Protein quantification was performed using a label-free approach, where peptides eluting from each LC run are detected as three-dimensional features – retention time versus signal intensity (extracted ion chromatogram, XIC) versus mass/charge – aligned and compared across runs, as previously described (Luber et al., 2010).

### Data Analysis

Analysis of data sets from decellularized ECM and CMs was loaded to Perseus software (version 1.6.2.1), and proteins only identified by site, reverse identification, and contaminants were excluded from the data. In the case of CM analysis, we also filtered out the proteins identified in nCM (LFQ≠0). LFQ intensities were log2 transformed, and the replicate samples were grouped. Proteins with less than two valid values in at least one group (for CM data) or in total samples (for ECM) were removed. For both cases, missing values were imputed from a normal distribution (with 0.3 spread and 1.8 downshift). Then, we achieved gene ontology (GO) term annotations (file main-Annot.homo sapiens.txt.gz), scatter plot, Pearson’s correlation, principal component analysis (PCA) and hierarchical clustering. A detailed experimental design is presented in Supplementary Figure 2. To evaluate proteins or genes differentially expressed (DE) during cardiac differentiation, we performed a two-sample test at Perseus using the no imputed data and comparing each differentiation time point with its respective previous time point.

Investigation of protein–protein interactions and functional enrichment GO analysis of DE proteins were performed with STRING database version 10.5 (Szklarczyk et al., 2017). Network lines represent the protein interaction score, which was based on four “evidence channels” (interaction sources): databases, experiments, text mining, and coexpression (Szklarczyk et al., 2017). For visualization of data as a heatmap, we used the software Morpheus^1^.

### Antibody Array for Secretome Characterization

Identification of key growth factors and cytokines released during cardiac differentiation was performed with a custom label-based antibody array for the detection of 60 proteins (Supplementary Figure 1B) following the manufacturer’s protocol (Raybiotech, GA, United States). After sample dialysis, 7 mg of protein for each sample was incubated overnight with the array membranes followed by incubation with HRP-conjugated streptavidin. Luminescent spots were detected using an imaging system (L-PIX Chemi Express, Loccus), with time exposure varying from 20 to 40 s. Images were analyzed using ImageJ software (version 1.52), and pixel density was determined in each spot using the protein array analyzer plugin (Carpentier and Henault, 2010). Each array was measured independently three times, and the values obtained were aligned accordingly to array map and normalized (based on internal negative and positive controls). The analysis of protein presence during differentiation has considered and discounted the values eventually found on nCM (CM pixel intensity). Only the proteins with a pixel intensity ≥50 in at least one time point were considered to be positive.

The results were plotted and visualized on GraphPad Prism 7, and to determine differential expression along cell differentiation time points, normalized pixel intensity was log2 transformed, and the log2 fold change (log2FC) was calculated by comparing each time point with its preceding one.

## Results

### *In vitro* Cardiomyogenesis Shows Extensive Modulation of Protein Secretion and ECM Deposition During hESC Differentiation Induction

The *in vitro* cardiomyogenic differentiation protocol performed in this study includes three leading stages: (1) EB formation, (2) mesodermal specification, and (3) cardiac progenitor development (Figure 1A). To confirm the progress of cell differentiation throughout these stages, we evaluated the morphology and phenotype of the cells. Figure 1B shows representative images of EBs grown along the assay. The expression of CD56, used to indicate cells committed to the mesodermal lineage, was >50% on the cultured cell population at D3 of the protocol, reaching to 80% at D4 (Figure 1C). After the addition of a Wnt pathway inhibitor (XAV939) to the cell culture, >50% of the population expressed eGFP/Nkx2.5at D9, showing its commitment to the cardiac cell lineage (Figure 1C). Pulsating EBs were observed from D10 of the protocol, generating a cell population with approximately 60% cardiomyocytes (cTnT^+^ cells) at D15 (Figure 1C). Therefore, to characterize the proteins released by hESCs during cardiomyogenesis, the CM and the ECM were collected at four distinct time points during the cell differentiation protocol: D1, D4 (CD56^+^ cells at day 3 = 64.5 ± 10%), D8/9 (eGFP^+^ cells = 58.25 ± 6.5%), and D15 (cTnT^+^ cells = 53.2 ± 4.2%) (Supplementary Figure 3). Collected samples derived from different experimental replicates were characterized by MS and/or immunolabeling and then analyzed.

#### Characterization of Conditioned Medium

After the initial filtering and removal of proteins identified in the basal medium (nCM), we started the proteome analysis with a list of 239 proteins identified by MS. Then, log2-transformed LFQ values were grouped according to the time point of the CM collection (CMd1, CMd4, CMd8, and CMd15). Only the proteins identified in two or more replicates in at least one CM sample were considered valid, resulting in a total of 106 proteins (Supplementary Table 1). Of these proteins, 92 were annotated as “ECM,” “extracellular organelle,” “extracellular region,” or “extracellular space.” Note that we chose to include “extracellular organelles,” since it is known that the cells also secrete extracellular membrane vesicles, which have important roles in cell communication (Camussi et al., 2010).

**FIGURE 2:**
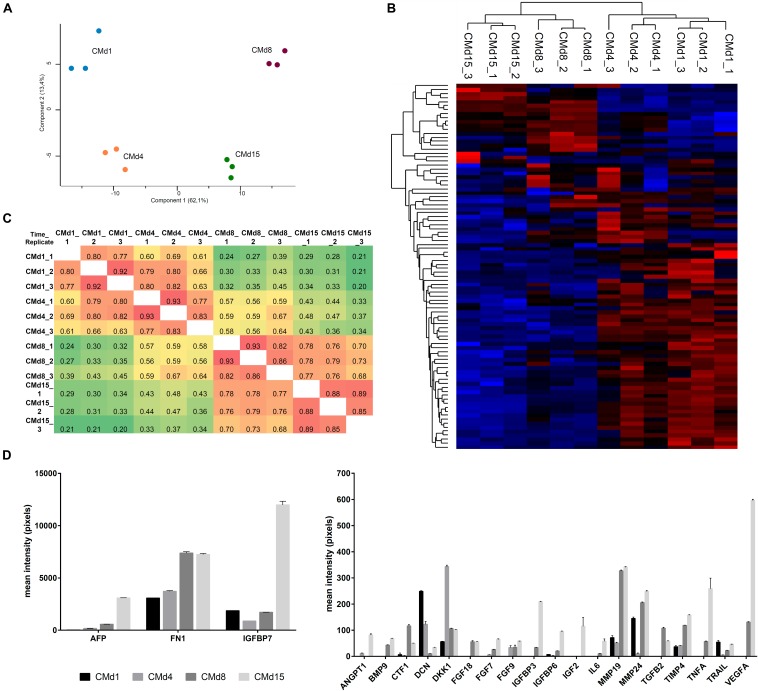
Characterization of CM obtained during cardiac differentiation of hESCs by mass spectrometry and antibody array. **(A)** Principal component analysis of CM replicates obtained at four time points of differentiation. **(B)** Heatmap of the hierarchical clustering of CM samples. High *z*-scores and low *z*-scores are represented in red and blue, respectively. **(C)** Pearson’s correlation comparing all the replicates with each other (higher correlation in red and lower correlation in green). **(D)** Graphs representing the intensity of proteins identified at CMs using an antibody array.

After replacing missing data values with imputed values from normal distribution (width 0.3 and downshift 1.8), PCA and hierarchical clustering of the samples showed that the three biological replicates of CM are similar and grouped mainly based on the time of differentiation (Figures 2A,B). The high Pearson correlation (>0.8) between replicates from different time points demonstrated high reproducibility (Figure 2C). We also observed from PCA that there is a separation between the CMs collected in the first days and those related to the more differentiated cell cultures (CMd1 × CMd4 and CMd8 × CMd15), which was also confirmed by lower Pearson correlation between CMs obtained from different time points (Figures 2A–C).

Analysis of CMs by a personalized antibody array resulted in the identification of 22 proteins (Supplementary Figure 1C). The proteins with the highest intensities were alpha-fetoprotein (AFP), fibronectin (FN1), and insulin-like growth factor-binding protein 7 (IGFBP7) (Figure 2D). Interestingly, with the exception of decorin (DCN) and dickkopf WNT signaling pathway inhibitor 1 (DKK1), which were identified in higher amounts in CMd1 and CMd4, respectively, all the other proteins appear with greater intensity mainly in the last days of the differentiation protocol (Figure 2D).

To identify modulated proteins in the secreted fraction throughout cell differentiation, we compared each cell differentiation time point with its preceding time point, as well as the CMd15 in relation to the initial time (CMd1), using a two-sample test at Perseus (applying the data without replace missing values). After this analysis, we selected proteins based on Student’s *t*-test significance and difference, as well as those identified exclusively in each of the CMs. Proteins with positive significance (+, *p*-value > 0.05) and with a *t*-test difference < 1 (downregulated) and >1 (upregulated) were included. The resulting list along with the antibody array results (−1 > log2 FC > 1) was used for the subsequent analyses. We could not identify any modulated protein when comparing CMd4 with CMd1, and only a few proteins were identified at one or another time point. However, comparing the CM from more differentiated cells, we observed 56 DE proteins in the CMd8 and 37 in the CMd15 (Supplementary Figure 4A). GO analysis of upregulated proteins in the CMd8 vs. CMd4 showed processes related to cell migration, proliferation, and even cardiovascular system development. On the other hand, analysis of upregulated proteins at CMd15 vs. CMd8 showed biological processes related to more general GO terms, such as organ morphogenesis (Supplementary Table 2).

Through comparison of CMd15 and CMd1 MS data, a large number of downregulated proteins were observed. However, considering the antibody array, only DCN was determined to be upregulated in CMd1. The association between the LC–MS/MS and antibody array results rendered GO terms for upregulated proteins in CMd15, which are related to biological processes, such as “ECM organization,” “regulation of cell migration,” “regulation of proliferation,” and “cardiovascular system development,” whereas the downregulated proteins indicated processes, such as “cellular localization” and “platelet degranulation” (Supplementary Table 2).

#### Characterization of Extracellular Matrix

**FIGURE 3:**
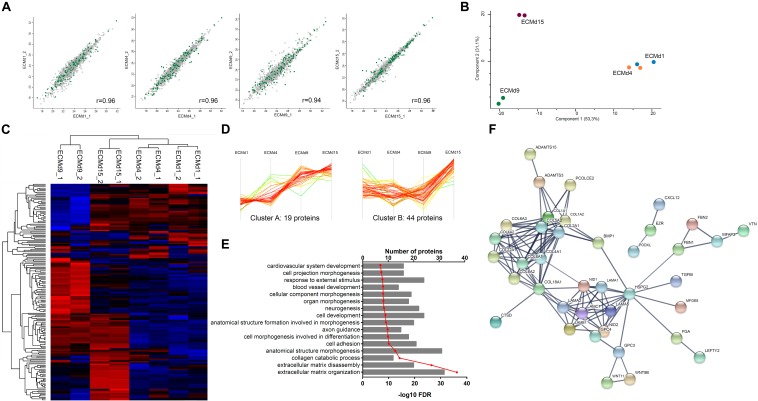
Characterization of extracellular matrix obtained from decellularized EBs during cardiac differentiation of hESCs. **(A)** Pearson’s correlation of ECM replicates. **(B)** Principal component analysis of all analyzed samples. **(C)** Heatmap representing the hierarchical clustering of ECM samples. High *z*-scores and low *z*-scores are represented in red and blue, respectively. **(D)** Two defined clusters showing a pattern with higher *z*-scores at ECMd15 (cluster A: gradual increasing over time; cluster B: increase from days 9 to 15). Clusters displayed the *z*-score values over analyzed times. **(E)** GO enrichment analysis for biological process of proteins present at clusters A and B. **(F)** Representative network of protein–protein interactions based on STRING analysis of proteins present at clusters A and B.

In an attempt to evaluate more specifically the ECM deposition during cell differentiation, we decellularized the EBs along the cardiac differentiation protocol. The DNA content in EBs was reduced by >90% compared to that in non-decellularized samples, confirming the efficiency of EB decellularization (Supplementary Figure 5A). Mass spectrometry data analysis (Supplementary Table 3) identified more than 2,000 proteins in the decellularized EBs with a very high Pearson correlation between time-point replicates (Figure 3A). Most of the proteins detected were related to cytoplasm, organelles, and other cell parts (Supplementary Figure 5B). Next, we filtered those proteins annotated as “ECM,” “extracellular region,” or “extracellular space,” obtaining a list of 170 proteins (Supplementary Table 3). PCA and clustering analysis demonstrated that the sample replicates (ECMd1, ECMd4, ECMd9, and ECMd15) were grouped and that ECMd1 and ECMd4 are much closer, whereas ECMd9 and ECMd15 are well-separated, evidencing important changes in these time points (Figures 3B,C).

Similar to the findings obtained for the CM, a small number of ECM proteins were regulated between ECMd4 and ECMd1. However, 91 and 100 out of the 170 ECM proteins showed differential expression (+ significance, −1 ≥ *T*-test difference ≥ 1) in ECMd9 and ECMd15, respectively (Supplementary Figure 4B). In a comparison of the upregulated proteins observed between ECMd9 and ECMd4, many GOs were enriched, including “cardiovascular system development.” This GO term was also identified in the analysis of ECMd15-upregulated proteins in relation to ECMd9 (Supplementary Table 4). However, when investigating the proteins involved in the same GO terms in each time point, we observed that most of them are different (Supplementary Figure 5C), suggesting that those proteins in D9 may be involved in the commitment of cells for cardiac lineage, while at D15, the proteins may be more related to the final differentiation or maturation of cardiomyocytes. Moreover, upregulated proteins in the ECMd15 showed GOs related to collagen metabolic and catabolic processes and collagen fibril organization, as confirmed by increasing levels of different collagen types at D15 (Supplementary Table 4).

Using the hierarchical clustering analysis, we defined clusters with distinct patterns of protein expression during cardiac differentiation. Of the five defined clusters, two showed proteins with high expression in the ECMd15: one exhibited a gradual increase in the amount of protein during differentiation (cluster A), while the other showed an increase only from D9 to D15 (cluster B) (Figure 3C). Proteins identified in these two clusters showed relationships with “ECM organization and disassembly,” “cell adhesion,” and “blood vessel and cardiovascular development” (Figure 3D and Supplementary Table 4). Furthermore, STRING-based analysis showed predicted interplay between these proteins (Figure 3E). At these time points, we observed two main groups of proteins: collagens and laminins, which interact with each other. Additionally, HSPG2 was connected with different classes of proteins, including laminins, BMP1, and TGFBI (Figure 3F).

#### Combined Analysis of Protein Identifications in CM and ECM

**FIGURE 4:**
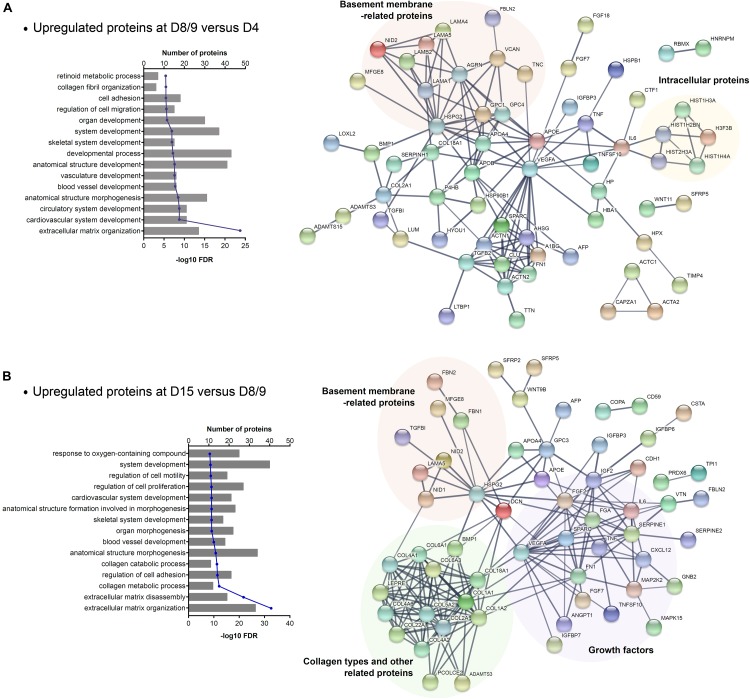
GO enrichment analysis of secretome (CM + ECM) proteins upregulated during late stages of cardiomyogenic differentiation. **(A)** Biological process related to proteins upregulated at days 8–9 (CMd8 and ECMd9) compared to the previous time point and the STRING network of these protein–protein interactions. **(B)** Biological process related to proteins upregulated at day 15 (CMd15 and ECMd15) compared to the previous time point and the STRING network of these protein–protein interactions.

To investigate the whole secretome of hESCs during cardiac differentiation, we associate the identified proteins that were modulated in CM and ECM samples throughout the differentiation time points. As the number of downregulated proteins was small, we focused on the upregulated proteins in an effort to identify networks and biological processes enriched in cardiac progenitor commitment (D8/9 vs. D4) and final cardiomyocyte differentiation (D15 vs. D8/9). A total of 80 and 76 proteins were upregulated in the secretome at D8/9 and D15, respectively. GO analysis of these selected proteins showed that as expected, “ECM organization” was the most significant term found. Furthermore, we also observed some general GO entries as “system development,” as well as GO terms specifically related to cardiovascular development (Figure 4 and Supplementary Table 5). Among the most significant GO terms related to the secretome at D15, biological processes linked to collagen metabolism and catabolism (Figure 4B) were highlighted, as also reported for ECMd15.

Moreover, through protein–protein interaction analysis, we observed that VEGFA and HSPG2 are central players in the networks, as they interact with many proteins. However, other proteins grouped in more specific interaction nodes (Figure 4). Considering the proteins secreted by cardiac progenitors (upregulated at D8/9), one of the interaction nodes that was identified is composed of basement membrane-related proteins, while some others are intracellular proteins (Figure 4A). In relation to the proteins modulated at D15, the network showed distinct groups: one with proteins related to basement membrane, another with collagen types and its related proteins, and a third that was composed of different types of growth factors (Figure 4B).

### Gene Expression Analysis of Extracellular-Related Proteins in Polysomal mRNA: Comparison to Cardiac Differentiation Secretome

Considering our previous data on polysome-bound mRNA sequencing during cardiomyogenic commitment of hESC (Pereira et al., 2018), we comparatively analyzed the Reads Per Kilobase Million (RPKM) values obtained from mRNA-seq with our current identifications in the secretome. After selection of the “protein_coding” genes, we filtered the genes found in at least two of the replicates in one or more cardiac differentiation time points of the protocol. Then, the respective “UniProt accession name” for each gene was added to the Perseus worksheet analysis to enable the GO annotation to be performed. Finally, only those genes related to ECM, extracellular region, or space were selected, generating a gene list with more than 1,800 genes (Supplementary Table 6). Interestingly, with only these 1,800 genes, it was possible to show reproducibility in the replicates, resulting in the grouping of the samples according to the degree of cell differentiation (Supplementary Figures 6A,B), which indicates the importance of extracellular signaling in cardiomyogenic differentiation.

Analysis of DE genes followed the same parameters as used for the secretome. The results showed a strong regulation in the transition of undifferentiated cell phenotype to mesodermal derivation (D4 × D1) and into cardiac progenitor commitment (D9 × D4) with fewer DE genes when comparing cells committed with differentiated phenotypes (D15 × D9) (Supplementary Figure 4C).

Throughout all the time points of the cell differentiation protocol, we identified several cell signaling mediators, such as FGFs, bone morphogenetic proteins (BMPs/GDFs), Wnt pathway proteins, interleukins, and chemokines. However, it was interesting to note that although many of these mediators belong to common multigene families, the specific genes up- or downregulated in each of the time points are different (Supplementary Figure 6C). Many of the genes upregulated in D4 and downregulated in D9 (D4 × D1 and D9 × D4) were in the Wnt pathway, which is a signaling pathway that modulates cardiac differentiation (Ueno et al., 2007). Although many of the identified Wnt genes were related to the commitment to mesodermal lineage, in D9, there was an increase in the expression of Wnt6, Wnt11, and Wnt2. The analysis of regulated genes from the beginning until the end of the differentiation showed GO terms related to “collagen catabolic process” (Supplementary Table 6); however, at each time point in the protocol, different types of collagen and members of the ADAMTS family (comprising disintegrin and metalloproteinase with thrombospondin motifs) were expressed.

**FIGURE 5:**
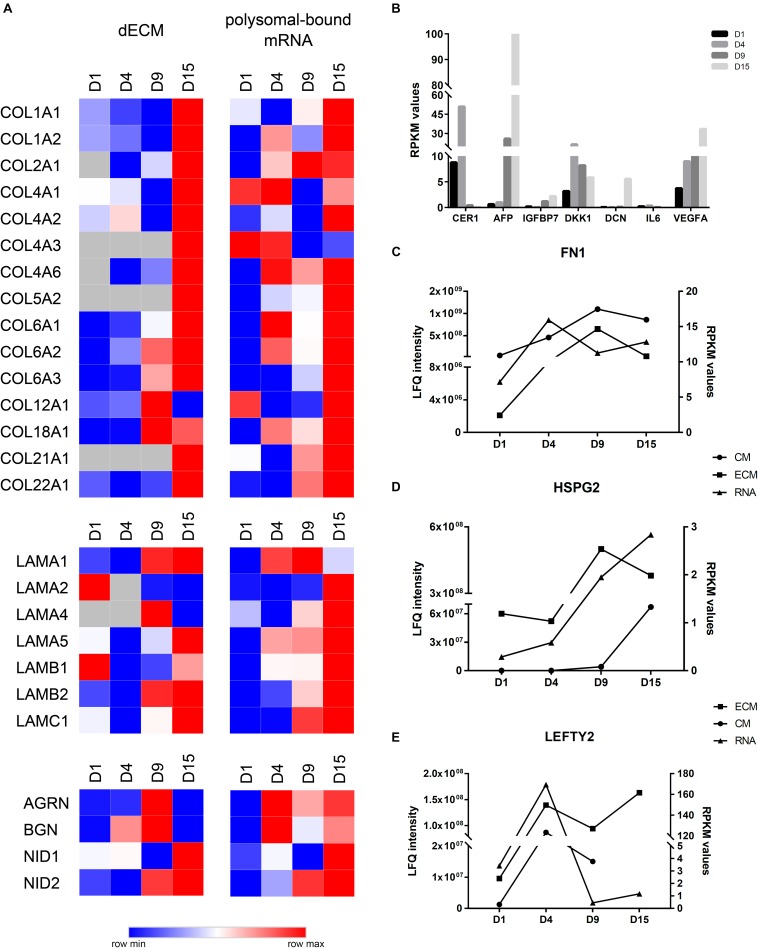
Comparison of ECM and CM results with polysomal mRNA sequencing data. **(A)** Heatmap visualization of log2 LFQ intensities (ECM) or log2RPKM values (RNA) of selected proteins/genes during cardiomyogenic differentiation of hESCs. Higher log2 values are represented in red, and the lower log2 values are represented in blue. **(B)** RPKM values of polysomal mRNA data of selected genes identified at CMs. **(C)** Comparison of LFQ intensities of fibronectin (FN1), **(D)** perlecan (HSPG2), and **(E)** LEFTY2 identified at ECM and CM with RPKM values of polysomal mRNA data.

Initial comparison of polysome-bound mRNA data with our MS identifications in the CM and the ECM showed an increase in DE genes or proteins at D9. Additionally, the number of upregulated proteins/genes in the ECM and in the polysomal mRNA were higher than the downregulated ones (Supplementary Figure 4). Then, we compared RPKM and LFQ intensity values to evaluate whether particular genes and proteins displayed similar expression profiles (Figure 5). Based on these observations, we defined three different gene expression patterns: (1) correlated expression when mRNA and protein increased or decreased at the same differentiation time points; (2) regulated expression when mRNA RPKM value was higher at a given time point, but the protein appeared with greater intensity only at the subsequent time point; and (3) no correlated expression when mRNA and protein showed no apparent correlation or an inverse relation. In this regard, most collagen subtypes and laminin subunits identified in the decellularized ECM showed a high prevalence at D15 and were correlated, in most cases, with a greater RPKM value in polysomal mRNA. COL2A1, COL4A2, COL6A3, LAMB2, LAMC1, and nidogens (NID1 and 2) are examples of this profile (Figure 5A). Similarly, AFP and IGFBP7 (identified at D15) and DKK1 and CER1 (identified at D4) were among the proteins present at higher quantities in the CM and with a similar pattern compared with RPKM values on polysomal mRNAs (Figure 5B). Furthermore, some proteins were identified in both ECM and CM. Some of the proteins, such as FN1 and HPSG2, found in ECM, CM, and mRNA showed increased expression throughout cell differentiation in all assays (Figures 5C,D). Other proteins, such as LEFTY2, presented a different result: while LEFTY2 in the CM presented greater expression on day 4 (corroborating with the RPKM values), in the ECM, it appeared to be more intense in both D4 and D15 (Figure 5E).

Another interesting observation was those genes/proteins identified with regulated expression, e.g., AGRN, BGN, LAMA1, LEFTY1, COL4A6, and COL18A1 (Figure 5A), which may indicate a different level of regulation. On the other hand, some other genes exhibited no correlation between RPKM values and protein intensities, as observed with IL6 and DCN found within the antibody array (Figures 2D, 5B).

In order to verify our results in other cardiac differentiation model, we select some proteins to analyze its expression throughout qPCR or immunostaining in monolayer cardiac differentiation protocol (Lian et al., 2013) (Supplementary Material and Supplementary Figure 7A), using not only the HES3 NKX2-5^eGFP/w^ lineage but also the H1 hESC. Initially, we confirmed the expression of markers such as Brachyury in the mesoderm stage (day 1) and the increased Nkx2.5 expression on day 9 for both hESC lineages (Supplementary Figure 7). From day 10 we observed contracting regions, which increased on day 15 (Supplementary Videos 1, 2); at this time-point we verified the higher expression of cTnT and cTnI in D15 monolayer in comparison with undifferentiated cells (Supplementary Figure 7). Then, our analysis confirmed the higher expression of CER1 on day 1 cells (comparative to D4 at EBs cardiac differentiation protocol), as well as increased expression of FN1, COL1A1, HSPG2, IGFPB7, VEGFA, and AFP at the end of the monolayer cardiomyogenic differentiation protocol (Supplementary Figure 8).

## Discussion

Cellular commitment throughout cardiomyogenesis comprises several cell differentiation steps, which are regulated by a network of cell signaling pathways (intercellular communication, extracellular milieu guidance, and transcription factor modulation) that control the specification and maturation of cardiac cells (Murry and Keller, 2008; Van Vliet et al., 2012). In an attempt to mimic the microenvironment and the cellular interactions during cardiomyogenesis, we employed a three-dimensional (EB culture) cardiac differentiation protocol using hESCs. Our cell differentiation protocol reached a percentage of cTnT^+^ cardiomyocytes approximately 60% from the total cell population after 15 days in culture, similar to that previously reported (Kattman et al., 2011; Pereira et al., 2018; Wolling et al., 2018). Although the presence of other cell types cannot be dismissed, our previous RNAseq data showed low expression of genes related to other cell lineages (Pereira et al., 2018). Furthermore, considering that our goal was to characterize the secretome during cardiac cell commitment and based on the knowledge that the presence of other cell types (e.g., endodermal cells and cardiac fibroblasts) enable the secretion of key factors that influence cardiomyogenesis, cell proliferation, and behavior (Fountoulaki and Dagres, 2015), we maintained this cell differentiation approach.

The hESC secretome during *in vitro* cardiac differentiation was characterized based on proteins identified in the CM and the ECM. Considering MS and antibody array approaches, we identified approximately 100 proteins in the CM, in which extracellular space-related proteins were enriched. Regarding ECM characterization, EBs were initially submitted to a decellularization protocol to enrich ECM proteins on samples. MS analysis of decellularized EBs allowed the identification of approximately 2,000 proteins, although only 170 were associated with the extracellular space. This identification pattern, considering the high presence of cellular contaminants in decellularized samples, is well-described in the literature (De Castro Brás et al., 2013; Calle et al., 2016), including in our group report (Robert et al., 2017), and is a challenge in the ECM research field (Chang et al., 2016). Nevertheless, considering that ECM proteins constitute approximately 1–1.5% of the mammalian proteome (not considering splicing variants and some associated proteins), this approach is recommended to allow ECM identifications (Hynes and Naba, 2012; Hynes, 2014). Furthermore, none of the previous studies performed a deeper proteomic characterization of decellularized EBs; only immunofluorescence for some ECM proteins have been shown in the literature (Nair et al., 2008; Goh et al., 2013; Sart et al., 2014). Our analysis associating identified proteins in CM and ECM (CM + ECM) showed an important modulation of the secretome throughout the differentiation stages, which involved many proteins related to signaling pathways (TGF, Hedgehog, Wnt, and others).

It is important to point out that our 3D *in vitro* model of cardiac differentiation resulted in data (including our previous RNAseq and the present secretome analysis) that were confirmed in monolayer cardiac differentiation (Supplementary Figures 7, 8) and corroborate the recent work of Wolling et al. (2018). The authors have analyzed the cardiac secretome over seven time points of monolayer cardiac differentiation and identified more than 400 secreted proteins altered during its differentiation. They also showed the modulation of several extracellular components over time, emphasizing in agonists and antagonists of pathways previously related to cardiac differentiation (Wolling et al., 2018). Despite protocol variations, similarities with our results were related to the great difference found in the secretome composition of uncommitted cell stages in comparison with stages of more committed cells, and the expression profile for many of the identified proteins (Supplementary Figure 9). Besides, our analysis focused in gene regulations throughout cell differentiation stages, comparing each time point with its previous one, aiming to identify the DE proteins in the main cardiac differentiation commitment steps. This enabled us to find and suggests interestingly biological process and protein interactions related to each cell differentiation stage.

A potent inhibitor of the Wnt pathway (Niida et al., 2004), also capable of interacting with and regulating the MesP1 transcription factor (Mesoderm Posterior BHLH Transcription Factor 1) (David et al., 2008), Dkk1 was found with higher LFQ intensity values in the secretome of cells committed with the mesodermal lineage (D4) – a cell specification that needs the canonical pathway activated (Lindsley et al., 2006) – reducing its level in subsequent time points. This profile was in accordance with previously described for CM (Wolling et al., 2018) and with RNA-seq data (Liu et al., 2017; Pereira et al., 2018). Another Wnt family members identified in our secretome results were Wnt9b at ECMd15 and Wnt11 at ECMd9 and ECMd15. These two proteins were identified with increased RPKM values during differentiation (Supplementary Figure 6), and it is possible that at these time points, a greater amount was complexed to the ECM. Wnt9b was able to influence epicardial development (Wang et al., 2005; Duchemin et al., 2017), although no further influence on cardiac development has been reported. Additionally, Wnt5a and Wnt11 were implicated in stimulating the non-canonical Wnt pathway, helping to promote cardiac differentiation through inhibition of β-catenin activity (Abdul-Ghani et al., 2011; Cohen et al., 2012; Bisson et al., 2015; Mazzotta et al., 2016). These findings indicated that endogenous molecules that activate or inhibit the Wnt pathway are secreted to control and induce the process of mesodermal and cardiac differentiation.

In parallel, some antagonists of BMP or Nodal pathways, as Cerberus 1 (CER1) (Piccolo et al., 1999) and the left–right determination factors 1 and 2 (LEFTY1-2) (Chen and Shen, 2004) were also found in our analysis. CER1 is a cytokine that was found with greater expression at D4 (both in CM and in polysomal mRNA) and besides it function as an antagonist of BMP and Nodal pathways, it is essential for cardiac progenitor commitment, leading to the induction of Baf60c, a chromatin remodeling factor which, in turn, affects the expression of cardiac transcription factors (Cai et al., 2013). In our data, LEFTY1 was identified with low expression values during differentiation. On the other hand, LEFTY2 was identified in CM, ECM, and polysomal mRNA with greater expression on D4, although in ECM, its level increased again on D15. Considering that LEFTY2 is more highly expressed in lateral plate mesoderm in mouse embryos (Meno et al., 1996; Kosaki et al., 1999), the presented data corroborate with the previously described findings but during *in vitro* differentiation.

As expected, we also found some TGFβ family members in our secretome analysis. One of these proteins was TGFβ2, which was found with higher expression at CMd8 and in polysomal mRNA from D9. It is important to point out that TGFβ2 deletion causes cardiac malformations in mice, possibly by alterations in epithelial-to-mesenchymal transition (Azhar et al., 2003, 2009). In addition, in mouse heart development, TGFβ2 mRNA is expressed at high levels in potential myocardial progenitor cells and is reduced later in cardiomyocytes (Dickson et al., 1993). Moreover, this gene was downregulated in adult CPCs compared with young CPC (Walravens et al., 2018). These findings suggested that TGFβ2 was secreted by cardiac progenitors and induced cardiomyogenesis in our cell differentiation protocol.

Other interesting identifications in our analysis were related to IGFBPs, which represent essential signals during embryo development by regulating the circulation and presence of IGF in tissues (Duan et al., 2010). Wolchinsky et al. (2014) have shown that the expression of IGFBP7, also known as angiomodulin (ANG), increased along the cardiomyogenic differentiation of murine ESCs and that its silencing reduced the expression of cardiomyogenic markers in these cells. Our data corroborate this finding, with IGFBP7/ANG being identified in the CMs at all time points, but with the greatest expression being observed in differentiated cells.

Considering the importance of ECM proteins during development and their role in storing and presenting secreted growth factors to cell membrane receptors (Rozario and DeSimone, 2010), one of the differentials of our work was to carry out the decellularization of EBs and the characterization of ECM throughout the differentiation. The literature has already explored the role of many ECM proteins during heart development and in pathological, remodeling, and tissue repairing conditions (Lockhart et al., 2011; Rienks et al., 2014; Williams and Black, 2015; Frangogiannis, 2017; Nielsen et al., 2017), but the ECM composition during *in vitro* cardiac differentiation was less investigated. In our analysis, we demonstrated that the ECM proteins showed a temporal modulation and, as in CM, we observed differences in ECM profile from cells at the beginning of cell differentiation process from that committed ones. Interestingly, an important difference was also verified between ECMd9 and ECMd15, which was related, for example, to collagen metabolism and catabolism, indicating a maturation of the ECM itself in D15. The collagens are essential in the heart, and their presence increases throughout heart development (Williams and Black, 2015), a pattern that was mimicked in our assays. FN1, an essential ECM glycoprotein linked to mesendodermal and cardiovascular cell commitment (Cheng et al., 2013; Chen et al., 2015), was found during all stages of cardiomyogenic differentiation. Interestingly, the amount of this protein was higher on day 8/9, while at the mRNA level, FN1 was more heavily expressed on D4. It is possible that the FN underwent posttranslational modifications until it was effectively secreted and dimerized together with other ECM proteins.

Likewise, some of the proteins identified in the EBs called attention for their role in controlling the bioavailability of growth factors, such as Wnt and BMP ligands. Among these proteins, we observed biglycan (BGN), a member of the small leucine-rich repeat proteoglycans (Moreno et al., 2005; Berendsen et al., 2011; Ye et al., 2012); fibrillins 1 and 2 (Nistala et al., 2010; Wohl et al., 2016); and glypicans (GPCs 1–6) (Sakane et al., 2012; Capurro et al., 2014). We also noticed the presence of important components of the basement membrane, such as collagen IV, laminins, HSPG2, and agrin (AGRN), found in almost all the analyzed time points. The presence of the basement membrane components and its correct assembly is essential for tissue development, including the heart (Hollfelder et al., 2014). As an example, it was previously suggested that laminin could bind Ca^+2^, regulating its concentration in the extracellular medium and consequently modifying cardiomyocyte electrical properties (Yang et al., 2014). Additionally, the components of the basement membrane could facilitate the formation of sarcomeres (Yang et al., 2015). Furthermore, the basal membrane protein AGRN was reported to stimulate the proliferation of primary culture cardiomyocytes and the differentiation of pluripotent stem cells, although it is also capable of delaying their maturation (Bassat et al., 2017). In our data, AGRN was found in greater amounts in ECMd9 and with reduced expression in ECMd15, possibly regulating the balance of differentiation induction into cardiac progenitors at D9 and the cellular maturation at D15.

Previously, data analysis of polysome-bound mRNAs obtained during *in vitro* hESC cardiac differentiation highlighted, among the upregulated genes, the “ECM organization” as one of the most significant biological processes, occurring mainly at mesoderm commitment (D4) and final differentiation (D15) (Pereira et al., 2019). Additionally, it was demonstrated that these genes were possibly posttranscriptionally regulated (Pereira et al., 2019). Comparison between global protein LFQ intensities and RPKM values of polysomal mRNA further indicates that these proteins undergo some level of regulation.

Taken together, our study highlights important microenvironmental signaling patterns that may modulate the *in vitro* cardiomyogenic differentiation of hESCs. The main change seems to occur in the commitment of cardiac progenitors (between D4 and D9); after that (D15) the secretome profile shows fewer significant changes, with the exception of ECM, which complexity increased notably. Moreover, among the observed patterns, we highlight some proteins such as IGFBP7, FN1, HSPG2, as other members of the basal lamina, and combinations of collagen types that were expressed at different levels according to the *in vitro* cardiac differentiation stage. Further studies must be carried out to investigate the role of these proteins in hESC differentiation and, specifically in relation to ECM proteins found here, if the combined use of them could promote a greater cardiomyogenic induction and/or cardiomyocyte maturation.

## Conclusion

Our characterization of the hESC secretome during cardiac differentiation, using both the CM and the ECM, allowed the depiction of microenvironmental signaling that may regulate cardiomyogenesis, interconnecting signaling forms, soluble factors, and ECM proteins. With respect to the ECM, our research mainly contributed to the knowledge about the modulation of this complex milieu during cardiac commitment of hESCs. Furthermore, the knowledge generated in this study can also contribute to the development of new strategies for cardiac differentiation, the maturation of cardiomyocytes, and advances for future acellular therapies.

## Data Availability Statement

Publicly available datasets were analyzed in this study. This data can be found here: NCBI Sequence Read Archive SRP150416 (2018).

## Ethics Statement

The studies involving human participants were reviewed and approved by the Comitê de Ética em Pesquisa do Instituto Oswaldo Cruz – Fiocruz/IOC. The ethics committee waived the requirement of written informed consent for participation.

## Author Contributions

AR and MS conceived the present work and wrote the manuscript. AR and IP performed the experiments. BD and MS supervised the findings of this work and helped to supervise the project. All authors made substantial intellectual contributions to the manuscript and approved the final version to be published.

## Conflict of Interest

The authors declare that the research was conducted in the absence of any commercial or financial relationships that could be construed as a potential conflict of interest.

## Footnotes

1 https://software.broadinstitute.org/morpheus
