# Supplementary material for: Secretome Analysis Performed During in vitro Cardiac Differentiation: Discovering the Cardiac Microenvironment
Source: Front Cell Dev Biol. 2020 Feb 7;8:49. doi: 10.3389/fcell.2020.00049 (PMC7025591; doi:10.3389/fcell.2020.00049)
Supplement: Supplementary file 9 [file Data_Sheet_1.pdf]

Supplementary Figures

A

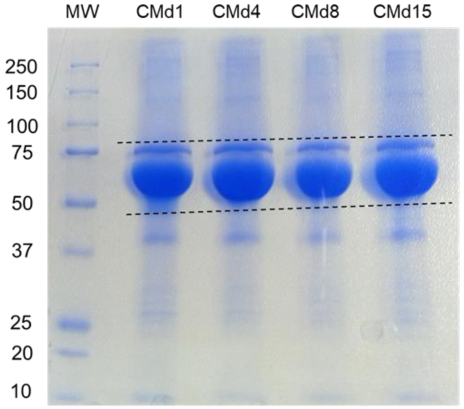

Protein names	Gene names	Unique peptides CMd1 (50-75)	Unique peptides CMd15 (50-75)
**Serum albumin**	**ALB**	**77**	**70**
**Serotransferrin**	**TF**	**45**	**47**
Hemopexin	HPX	15	14
Afamin	AFM	11	7
Haptoglobin	HP	8	7
Alpha-1B-glycoprotein	A1BG	8	8
Alpha-1-antichymotrypsin	SERPINA3	7	4
Alpha-1-antitrypsin	SERPINA1	3	3
Alpha-2-HS-glycoprotein	AHSG	3	4
N-acetylmuramoyl-L-alanine amidase	PGLYRP2	3	3

B

POS	POS	NEG	NEG	AFP	Angiopoetin-1	Angiostatin	APRIL	Biglycan	BMP-4	BMP-5	BMP-7	BMP-9	Cardiotrophin-1
POS	POS	NEG	NEG	AFP	Angiopoetin-1	Angiostatin	APRIL	Biglycan	BMP-4	BMP-5	BMP-7	BMP-9	Cardiotrophin-1
CD30ligand	CXCL14	CXCL16	Decorin	Dkk-1	EGF	ENA-78	Erytropoeitin	FGF-10	FGF-12	FGF-17	FGF-18	FGF-19	FGF-23
CD30ligand	CXCL14	CXCL16	Decorin	Dkk-1	EGF	ENA-78	Erytropoeitin	FGF-10	FGF-12	FGF-17	FGF-18	FGF-19	FGF-23
FGF-4	FGF-7	FGF-9	Fibronectin	Frizzled-1	GCP-2	GDF-15	GDF3	GRO	HB-EGF	HGF	IGFBP-1	IGFBP-3	IGFBP-5
FGF-4	FGF-7	FGF-9	Fibronectin	Frizzled-1	GCP-2	GDF-15	GDF3	GRO	HB-EGF	HGF	IGFBP-1	IGFBP-3	IGFBP-5
IGFBP-6	IGFBP-7	IGF-II	IL-6	Lumican	MMP-19	MMP-2	MMP-24	MMP-25	PDGF-BB	SDF-1	TGF-alpha	TGF-beta1	TGF-beta2
IGFBP-6	IGFBP-7	IGF-II	IL-6	Lumican	MMP-19	MMP-2	MMP-24	MMP-25	PDGF-BB	SDF-1	TGF-alpha	TGF-beta1	TGF-beta2
TIMP-4	TNF-alpha	TRAIL	Vasorin	VEGF	VEGI	VGF	VWF	BLANK	BLANK	BLANK	BLANK	BLANK	POS
TIMP-4	TNF-alpha	TRAIL	Vasorin	VEGF	VEGI	VGF	VWF	BLANK	BLANK	BLANK	BLANK	BLANK	POS

C

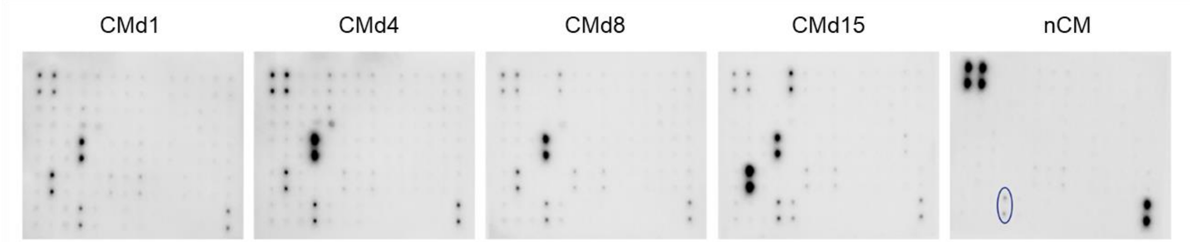

Supplementary figure 1: Analysis of conditioned medium obtained during cardiac differentiation. A) Representative image of 10% SDS-PAGE of CMs samples, indicating the region of 50-75 kDa (dotted line) which correspondes to the mass spectrometry protein identification in table (right). B) Map of proteins on array of custom antibody array. C) Representative images of array after CMs incubation and detection.

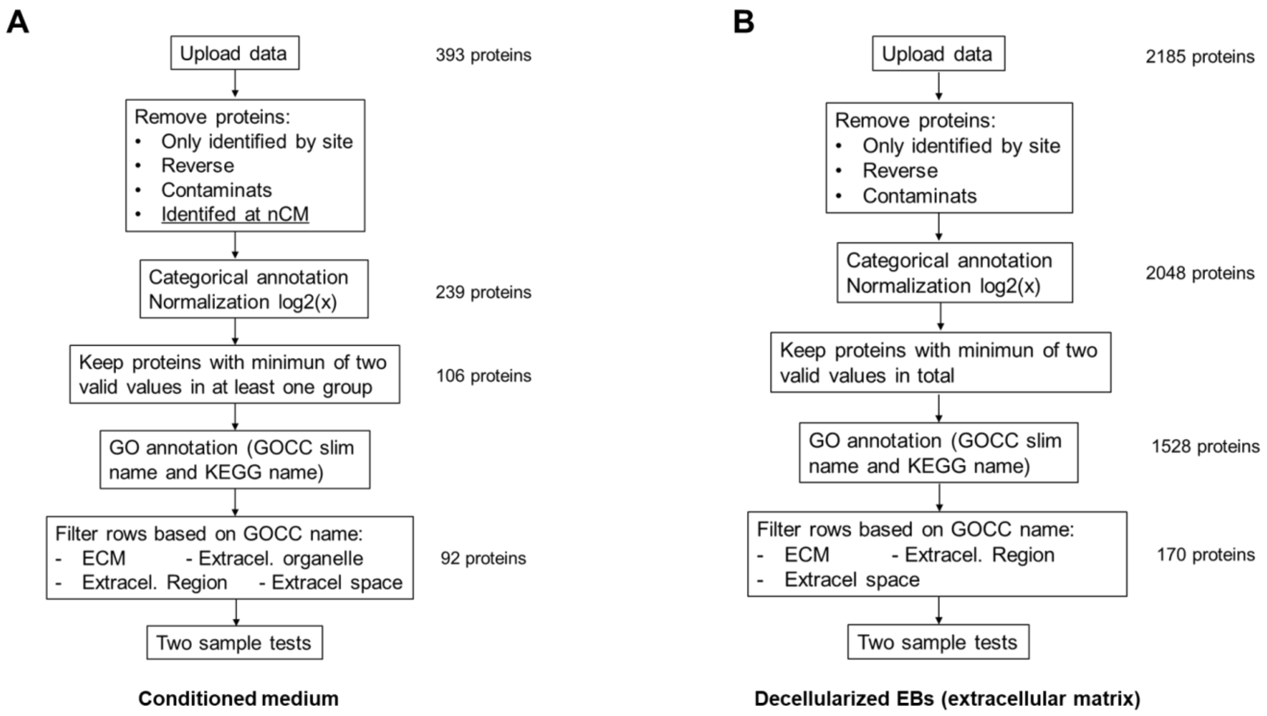

Supplementary figure 2: Analysis steps of mass spectrometry data, at Perseus, from CMs (A) and ECM samples (B).

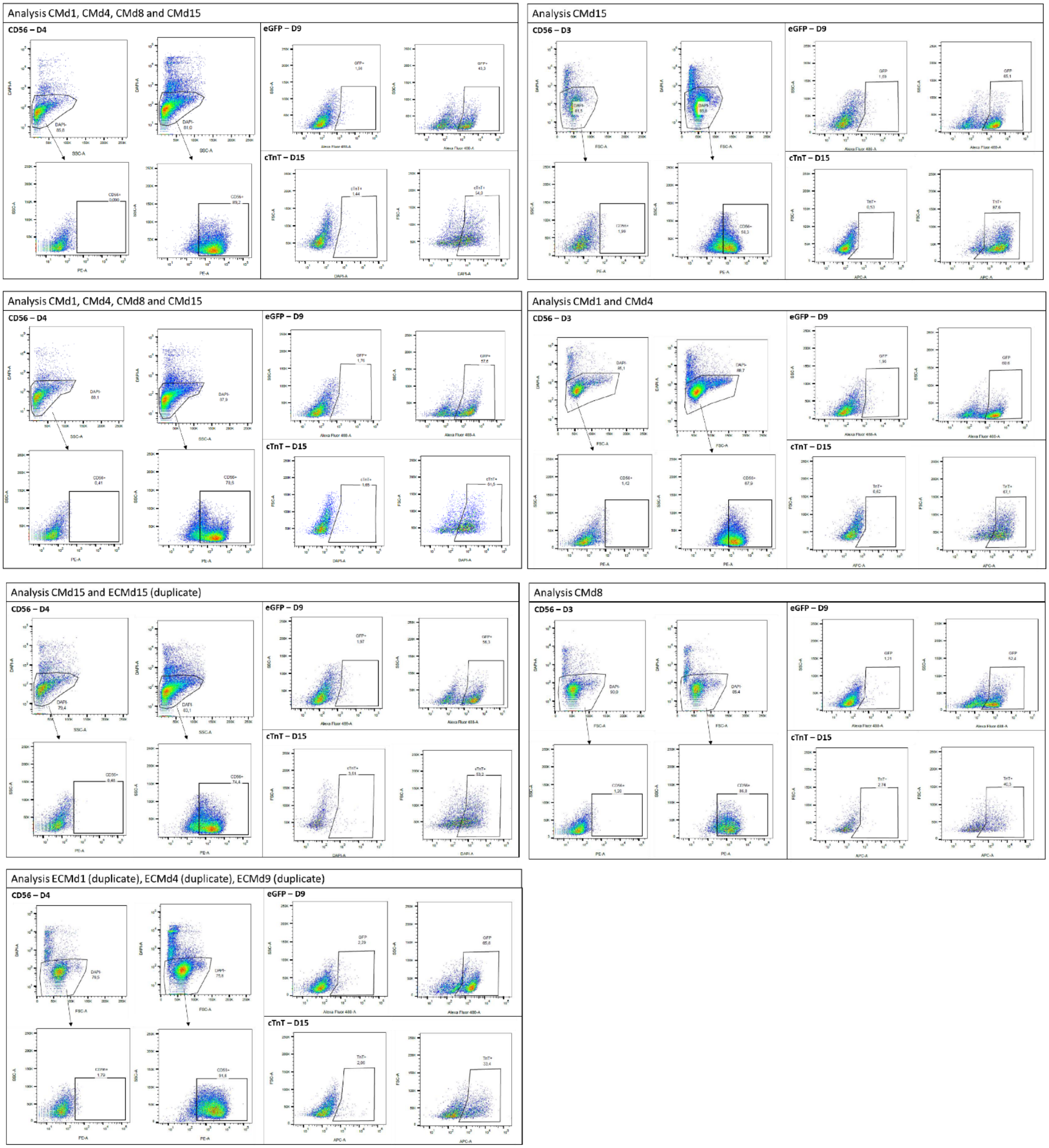

Supplementary figure 3: Representative dot plots of flow cytometry analysis of the following markers: CD56 (day 3), eGFP/Nkx2.5 (day 9) and cTnT (day 15) of all experiments used to collected CM or ECM. In the top of each frame/ experiment it is indicated which time-point (chosen based on the% of stained cells at the time-point) was analyzed by mass spectrometry or antibody array.

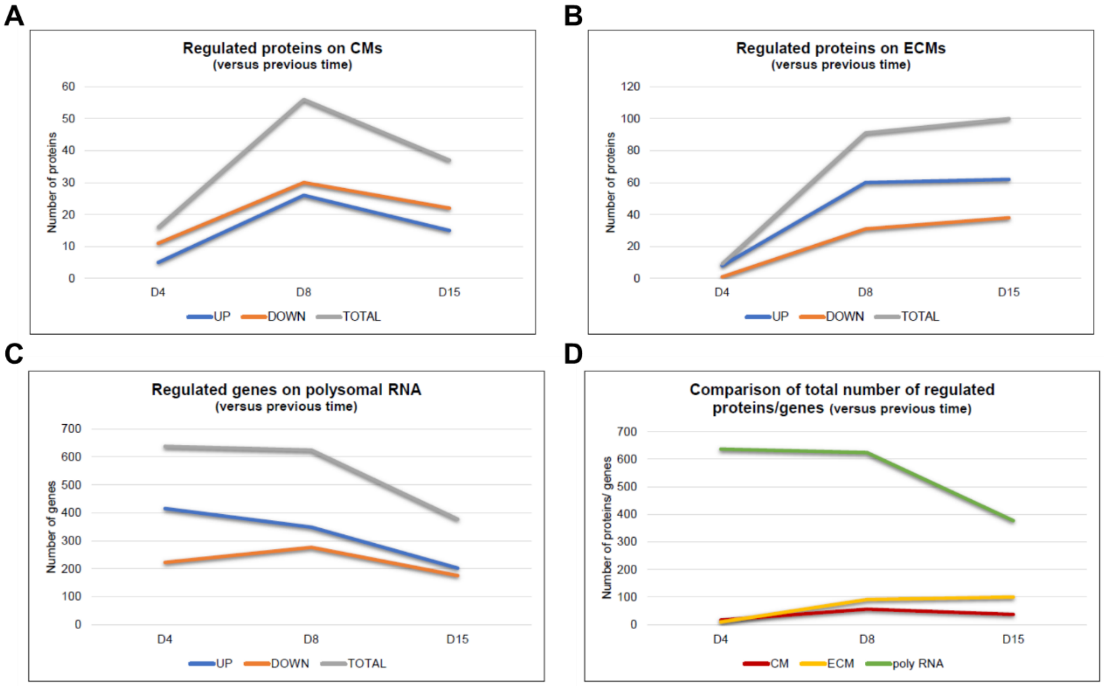

Supplementary figure 4: Number of proteins/genes differentially expressed (DE) during cardiac differentiation comparing each differentiation time-point with the preceding one. Indication of the number of regulated proteins at CMs (A) and at ECM (B), the number of regulated genes (C) and the comparison of total number of proteins/genes differentially expressed (DE) at CM, ECM and polysomal mRNA (D). Blue lines: upregulated proteins/ genes; Orange lines: downregulated proteins/ genes; Gray lines: total regulated proteins/ genes.

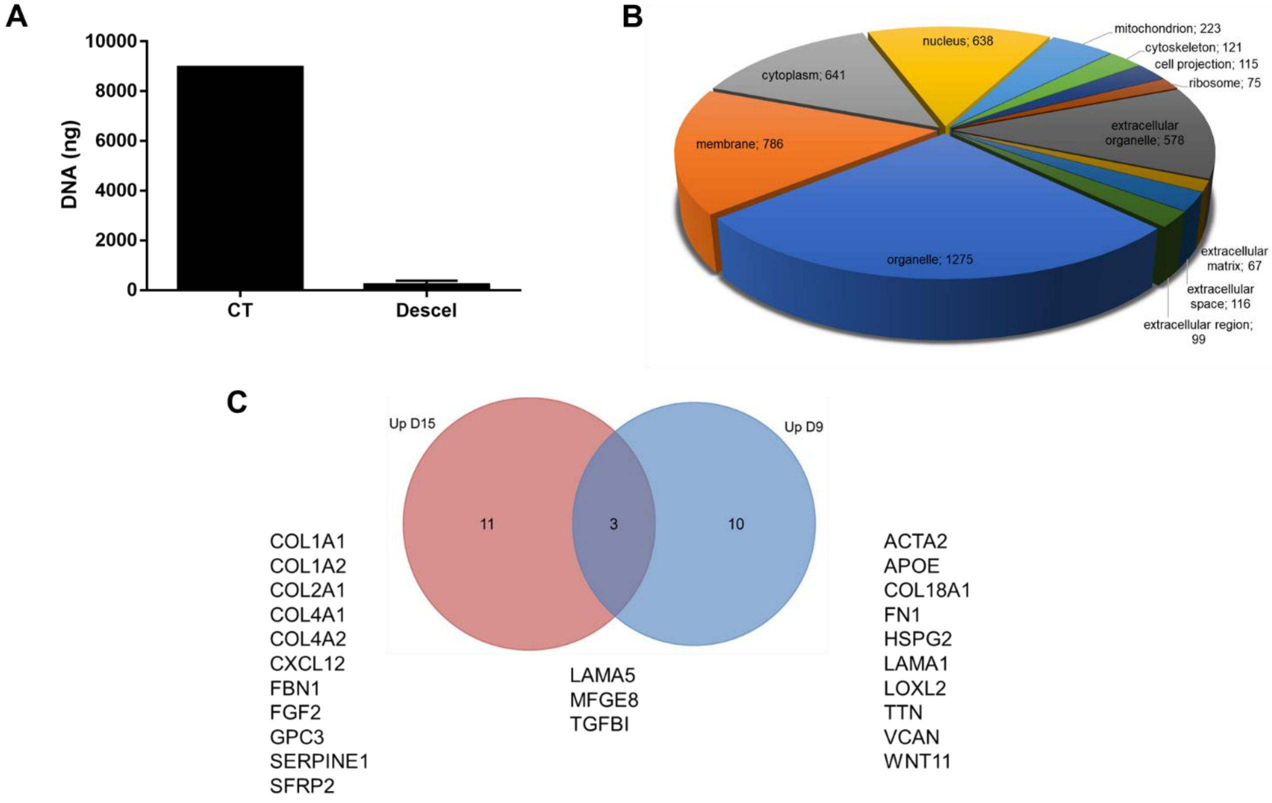

Supplementary figure 5: Analysis of decellularized EBs over cardiac differentiation. A) Quantification of DNA from decellularized and non decellularized EBs. B) GO annotation for cellular components at Perseus. C) Comparison of proteins related to the GO term “cardiovascular system development” identified in GO analysis of proteins upregulated at ECMd9 (up ECMd9) and ECMd15 (up ECMd15).

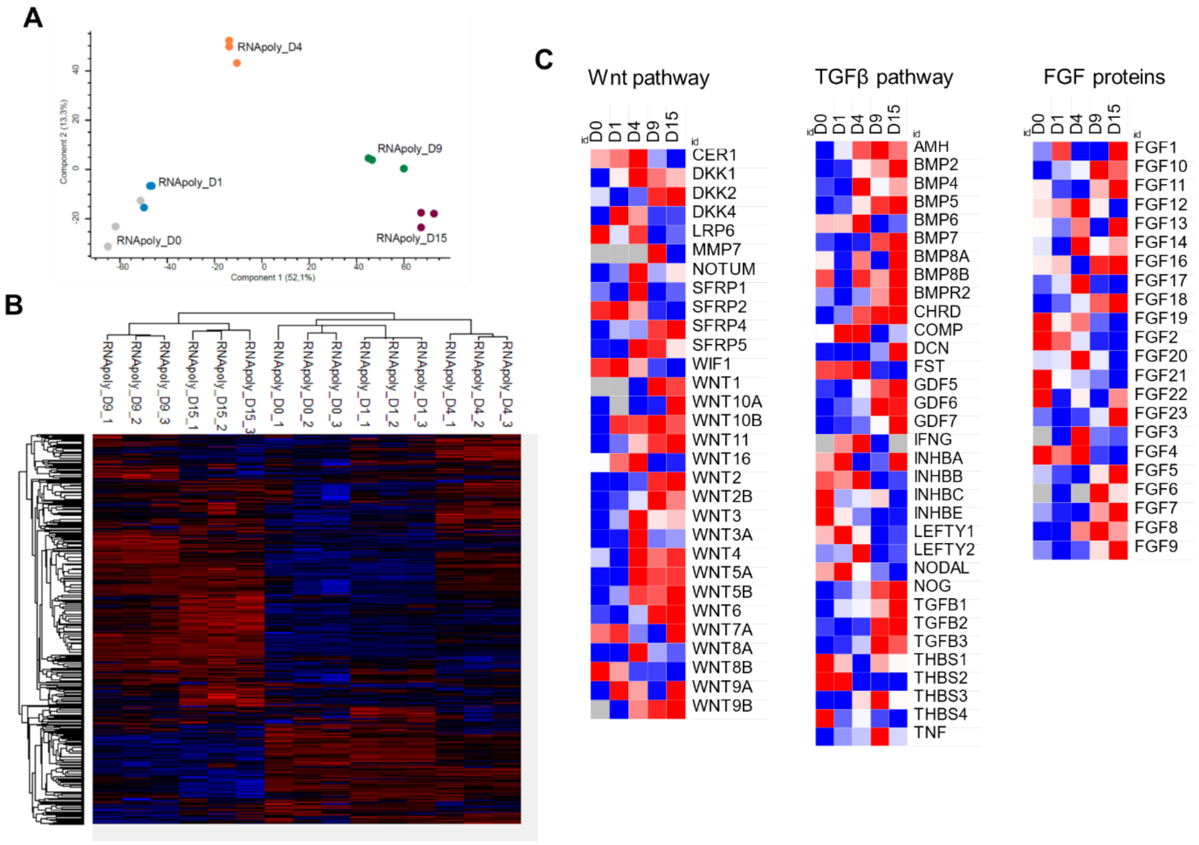

Supplementary figure 6: Analysis of polysomal mRNA data obtained during cardiomyogenic differentiation of hESC. A) Principal component analysis of data from polysomal mRNA sequencing obtained at days 0, 1, 4, 9 and 15 of cardiac differentiation. B) Hierarchical clustering of data from polysomal mRNA sequencing obtained at days 0, 1, 4, 9 and 15 of cardiac differentiation (high z-scores and low z-scores are represented in red and blue, respectively). C) Heatmap visualization of RPKM values of some genes related to Wnt (left), TGFb (middle) and FGF (right) pathways (high log2 values and low log2 values are represented in red and blue, respectively).

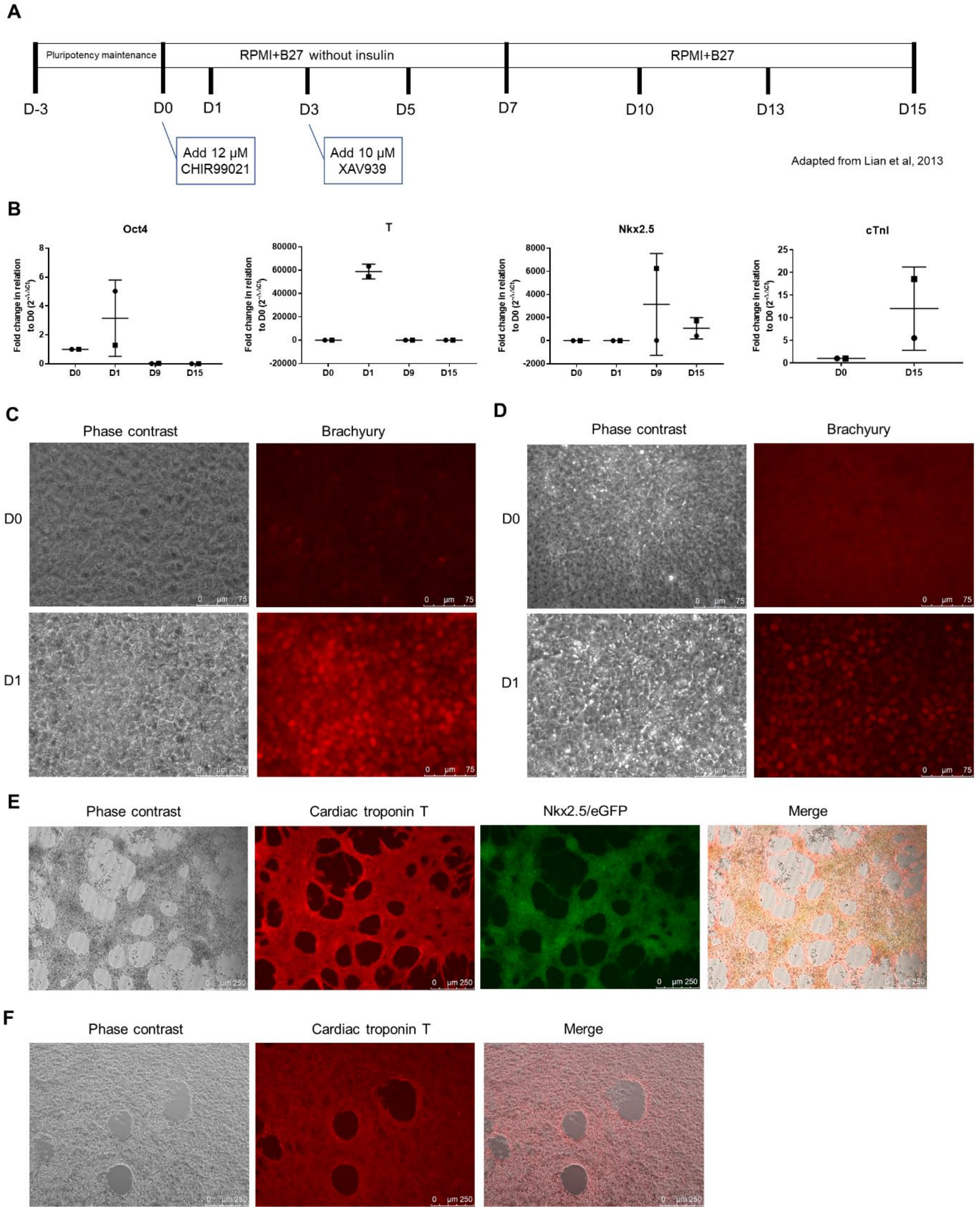

Supplementary figure 7: Monolayer cardiac differentiation of HES3 NKX2-5$^{eGFP/w}$ and H1 hESC lineages. A) Scheme of monolayer cardiac differentiation protocol. B) Relative expression ($2^{-\Delta\Delta Ct}$ method) of OCT4, Brachyury (T), Nkx2.5 and cTnI genes in cells lineages (circle represents the HES3 and the square the H1 lineage). Representative images of immunostaining for Brachyury at D0 and D1 in H1 (C) and HES3 (D) cells. Scale bar: 75 µm. Representative images of immunostaining for cTnT in monolayer of HES3 (E) (also possible verify eGFP expression) and H1 (F) cells. Scale bars: 250 µm.

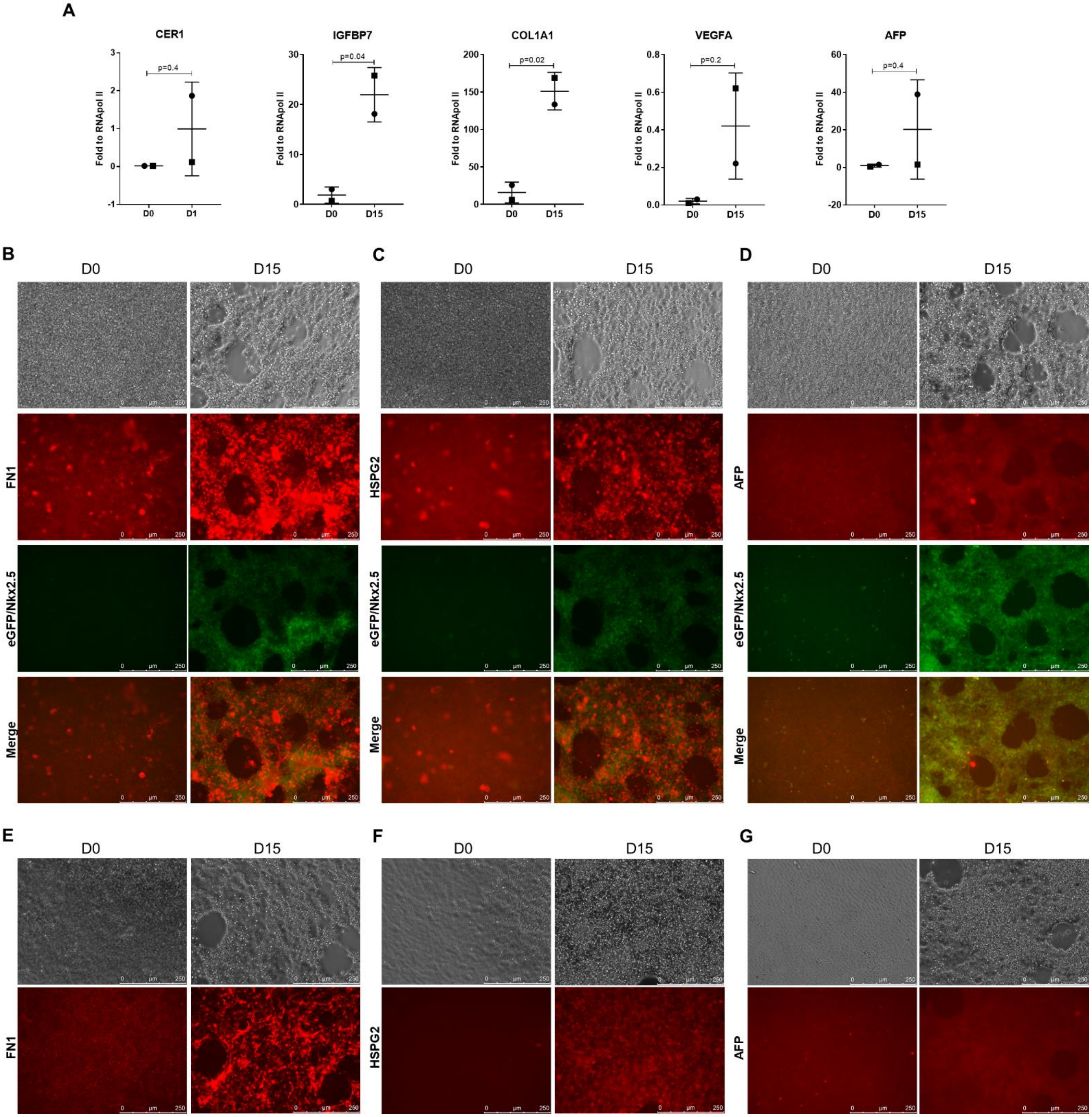

Supplementary Figure 8: A) Results of gene expression for CER1, IGFBP7, COL1A1, VEGFA and AFP represented by fold to RNApol II (circle represents the HES3 and the square the H1 lineage). Unpaired T-test statistical analysis. Representative images of FN1 (B), HSPG2 (C) and AFP (D) staining in HES3 NKX2-5$^{eGFP/w}$ cells at D0 and D15 of monolayer cardiac differentiation. It is also possible verify eGFP/Nkx2.5 expression. Representative images of FN1 (E), HSPG2 (F) and AFP (G) staining in H1 cells at D0 and D15 of monolayer cardiac differentiation. Scale bars: 250 µm.

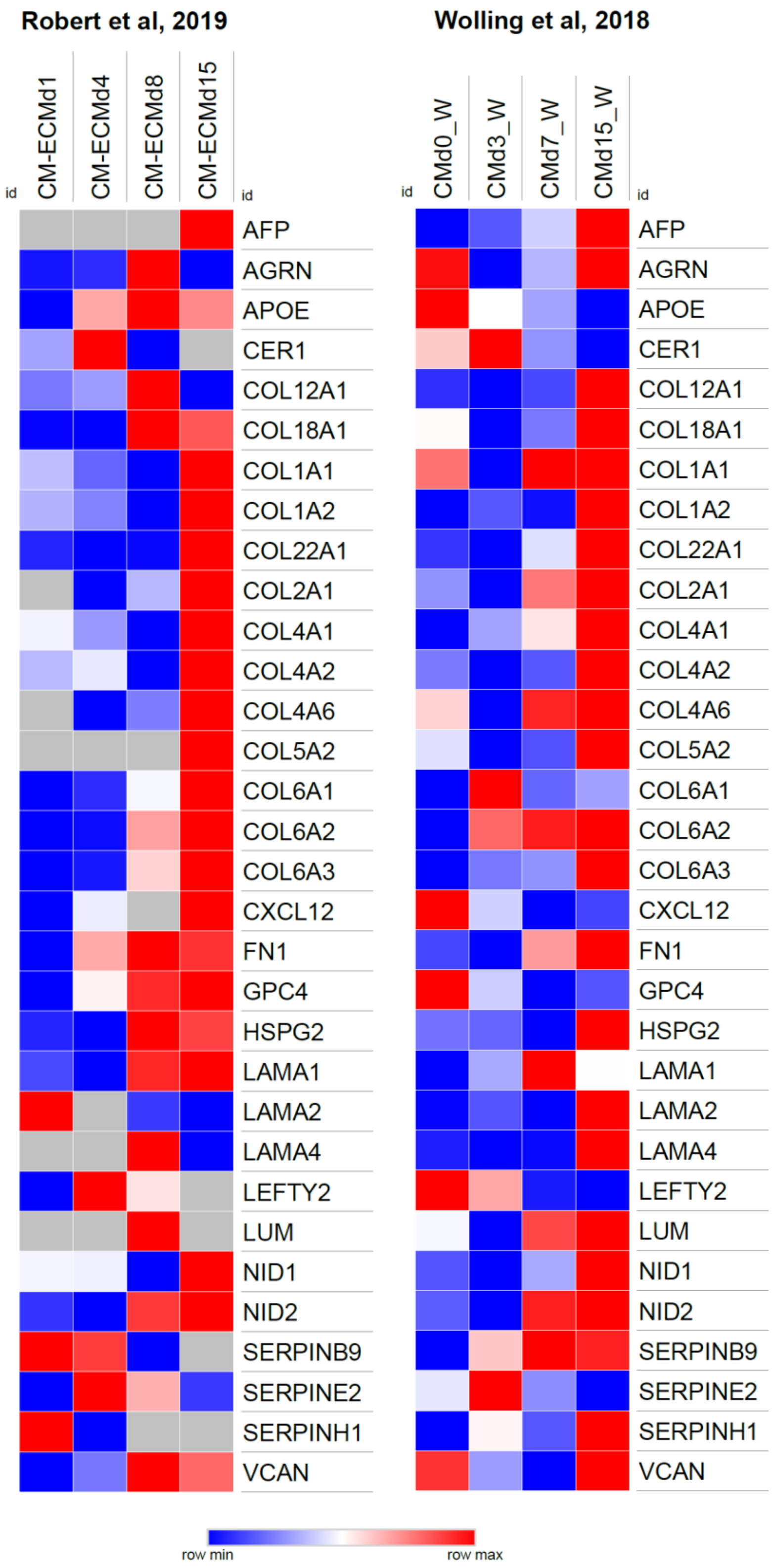

Supplementary figure 9: Heatmap comparing LFQ intensity data for some extracellular proteins from our work (left) with previously work from Wolling et al., 2018 (right).
