# Supplementary material for: Secretome Analysis Performed During in vitro Cardiac Differentiation: Discovering the Cardiac Microenvironment
Source: Front Cell Dev Biol. 2020 Feb 7;8:49. doi: 10.3389/fcell.2020.00049 (PMC7025591; doi:10.3389/fcell.2020.00049)
Supplement: Supplementary file 10 [file Data_Sheet_2.PDF]

frontiers

Supplementary Material

Monolayer cardiac differentiation protocol

HES3 NKX2-5$^{eGFP/w}$ lineage were dissociated from iMEF cultures and 1.5x10^{5} cells/well were plated into growth factor reduced Matrigel® Matrix (Corning) coated wells in 24-well dishes. On the other hand, H1 lineage were cultured in Matrigel® hESC-qualified Matrix (Corning) with mTeSR™ 1 (StemCell Technologies). After reached 70% of confluency, cells were dissociated using 0.25% trypsin/EDTA and 2.5x10^{5} cells/well were plated into growth factor reduced Matrigel® Matrix (Corning) coated wells in 24-well dishes.

Both hESCs lineages were maintained in pluripotency medium (hESC medium or mTeSR™ 1) until they were 100% confluent (ranged from two to three days). The protocol used followed the indications described by Lian et al., 2013 (Supplementary Figure 7a). At day 0, RPMI medium supplemented with B27 without insulin (RPMI+B27-insulin) and 12 µM CHIR99021 (Sigma-Aldrich) were added to the culture for 24h. At day 1, cells were maintained in RPMI+B27-insulin. On day 3, RPMI+B27-insulin supplemented with 10 µM XAV939 (Sigma-Aldrich) were added to the monolayer cultures, as described by Lian et al (2013). At day 5 the medium was exchanged for RPMI+B27-insulin. From day 7, the hESC were cultured in RPMI supplemented with B27 complete and the medium exchanges occurred every 3 days until day 15. On days 0, 1 and 15 monolayers were fixed with 4% paraformaldehyde and followed for immunofluorescence staining, based in a previously described protocol (Pereira et al., 2018).

RNA extraction and RT-qPCR

RNA was extracted using TRIzol™ reagent (Invitrogen™) and the cDNA reaction was performed using an ImProm-II™ Reverse Transcription System (Promega) according to the manufacturer's instructions. Samples were obtained from H1 and HES3 cells at different time-points during monolayer cardiac differentiation: day 0 (undifferentiated cells), day 1 (mesoderm committed cells), day 9 (cardiac progenitors) and day 15 (cardiomyocytes). cDNA amplification experiments were carried out with GoTaq® qPCR Master Mix (Promega), in a final volume of 10 µl. The RT-qPCR conditions followed the manufacturer's recommendations (Promega), using the LightCycler system (Roche). RT-qPCR experiments were performed in triplicate. The Cq results for each gene were normalized based on RNA polymerase II (RNApol II). The data were plotted as Fold to RNApol II or as relative expression ($2^{-\Delta\Delta Ct}$ method). The analyzed genes and the primer sequences are shown bellow:

Gene	Sequence (5'-3')	Amplicon (bp)	Reference
Oct3/4F	F: ATGCATTCAAACTGAGGTGCCTGC<br>R: AACTTCACCTTCCCTCCAACCAGT	192 bp	(Ye et al., 2013)

CER1	F: ACAGTGCCCTTCAGCCAGACT<br>R: ACAACTACTTTTTCACAGCCTTCGT	53 bp	(Lee et al., 2009)
COL1A1	F: GGCCATCCAGCTGACCTTCC<br>R: CGTGCAGCCATCGACAGTGAC	205 bp	(Aguiar et al., 2011)
Nkx2.5	F: TTAAGTCACCGTCTGTCTCCCTCA<br>R: ACCGACACGTCTCACTCAGCATTT	124 bp	(Ye et al., 2013)
VEGFA	F: CTACCTCCACCATGCCAAGTG<br>R: TGCGCTGATAGACATCCATGA	101 pb	(Schittini et al., 2010)
cTnT	F: TGCAGGAGAAGTTCAAGCAGCAGA<br>R: AGCGAGGAGCAGATCTTTGGTGAA	155 bp	(Ye et al., 2013)
cTnI	F: GGGGGCCCGGGCTAAGGAGTC<br>R: AGGGCAGGGGCAGTAGGCAGGAAG	183 bp	(Schittini et al., 2010)
Brachyury (T)	F: AAAGAGATGATGGAGGAACCCGGA<br>R: AGGATGAGGATTTGCAGGTGGACA	~108 bp	(Ye et al., 2013)
AFP	F: CAGCCACTTGTTGCCAACTC<br>R: GGCCAACACCAGGGTTTACT	123 bp	PrimerBlast
IGFPB7	F: CACTGGTGCCCAGGTGTACT<br>R: TTGGATGCATGGCACTCATA	240 bp	(Rao et al., 2014)
RNApol II	F: TACCACGTCATCTCCTTTGATGGCT<br>R: GTGCGGCTGCTTCCATAA	187 bp	(Aguiar et al., 2011)

References

Aguiar, A. M. De, Kuligovski, C., Costa, M. T. B. A. da, Stimamiglio, M. A., Rebelatto, C. L. K., Senegaglia, A. C., et al. (2011). Alkaline phosphatase-positive cells isolated from human hearts have mesenchymal stem cell characteristics. *Stem Cell Discov.* 1, 71–80. doi:10.4236/scd.2011.13008.

Lee, L. H., Peerani, R., Ungrin, M., Joshi, C., Kumacheva, E., and Zandstra, P. (2009). Micropatterning of human embryonic stem cells dissects the mesoderm and endoderm lineages. *Stem Cell Res.* 2, 155–162. doi:10.1016/j.scr.2008.11.004.

Lian, X., Zhang, J., Azarin, S. M., Zhu, K., Hazeltine, L. B., Bao, X., et al. (2013). Directed cardiomyocyte differentiation from human pluripotent stem cells by modulating Wnt/β-catenin signaling under fully defined conditions. *Nat. Protoc.* 8, 162–75. doi:10.1038/nprot.2012.150.

Pereira, I. T., Spangenberg, L., Robert, A. W., Amorín, R., Stimamiglio, M. A., Naya, H., et al. (2018). Polysome profiling followed by RNA-seq of cardiac differentiation stages in hESCs. *Sci. Data* 5, 180287. doi:10.1038/sdata.2018.287.

Rao, C., Lin, S.-L., Ruan, W.-J., Wen, H., Wu, D.-J., and Deng, H. (2014). High Expression of IGFBP7 in Fibroblasts Induced by Colorectal Cancer Cells Is Co-Regulated by TGF-β and Wnt Signaling in a Smad2/3-Dvl2/3-Dependent Manner. *PLoS One* 9, e85340. doi:10.1371/journal.pone.0085340.

Schittini, A. V, Celedon, P. F., Stimamiglio, M. A., Krieger, M., Hansen, P., da Costa, F. D. A., et al. (2010). Human cardiac explant-conditioned medium: soluble factors and cardiomyogenic effect on mesenchymal stem cells. *Exp. Biol. Med.* 235, 1015–1024. doi:10.1258/ebm.2010.010003.

Ye, L., Zhang, S., Greder, L., Dutton, J., Keirstead, S. A., Lepley, M., et al. (2013). Effective Cardiac Myocyte Differentiation of Human Induced Pluripotent Stem Cells Requires VEGF. *PLoS One* 8. doi:10.1371/journal.pone.0053764.

Supplementary legends

Supplementary video 1: Video of day 15 beating monolayer of HES3 NKX2-5$^{eGFP/w}$.

Supplementary video 2: Video of day 15 beating monolayer of H1 hESC lineage.

Supplementary table 1: Mass spectrometry analysis (Perseus software) of conditioned medium obtained during EBs cardiac differentiation.

Supplementary table 2: Gene ontology analysis (gProfiler) of differentially expressed proteins identified comparing one time point with its previous one in conditioned medium samples.

Supplementary table 3: Mass spectrometry analysis (Perseus software) of decellularized extracellular matrix obtained during EBs cardiac differentiation.

Supplementary table 4: Gene ontology analysis (gProfiler) of differentially expressed proteins identified comparing one time point with its previous one in decellularized extracellular matrix samples.

Supplementary table 5: Combined gene ontology analysis (gProfiler) of differentially expressed proteins identified in conditioned medium and decellularized extracellular matrix samples.

Supplementary table 6: Analysis of polysomal-bound mRNA sequencing data (previously published; Pereira et al., 2018) using Perseus software.
